# Genome-Wide Identification and Characterization of the Class III Peroxidase Gene Family in Radish (*Raphanus sativus*) with Insights into Their Roles in Anthocyanin Metabolism

**DOI:** 10.3390/ijms26135917

**Published:** 2025-06-20

**Authors:** Zihao Wei, Weimin Fu, Xianxian Liu, Wenling Xu, Lichun Chang, Chen Liu, Shufen Wang

**Affiliations:** Shandong Key Laboratory of Bulk Open-Field Vegetable Breeding, Ministry of Agriculture and Rural Affairs Key Laboratory of Huang Huai Protected Horticulture Engineering, Institute of Vegetables, Shandong Academy of Agricultural Sciences, Jinan 250100, China; zihao.wei@foxmail.com (Z.W.);

**Keywords:** class III peroxidase, radish, anthocyanin, genome-wide, transcriptome, expression analysis

## Abstract

Class III peroxidases (PODs) are plant-specific enzymes that play crucial roles in plant growth, development and responses to stress. However, the *POD* gene family in the radish (*Raphanus sativus* L.) has not been comprehensively investigated to date. In this study, a total of 95 *RsPODs* were identified in the radish genome, which were classified into six subgroups based on a phylogenetic analysis. The gene structures and conserved motifs of the *RsPODs* were highly conserved within each subgroup. An intraspecific collinearity analysis revealed 7 tandem and 40 segmental duplication events. An expression analysis across diverse tissues and developmental stages demonstrated that the *RsPODs* were functionally involved in radish development. Using a chimeric-colored radish mutant, this study revealed significantly higher POD activity in the green tissues compared to purple tissues. Through transcriptome sequencing, two *RsPOD* genes (*RsPOD14* and *RsPOD28*) were identified as candidate genes related to the anthocyanin metabolism. Our study provides a genome-wide perspective on the *RsPOD* genes in the radish and highlights their potential roles in the anthocyanin metabolism. These findings establish a critical foundation for future research aimed at uncovering the functional roles of specific *RsPOD* genes, with a particular emphasis on elucidating the molecular mechanisms that regulate anthocyanin degradation in the radish.

## 1. Introduction

Peroxidases (EC 1.11.1.X) are a ubiquitous class of enzymes found across all domains of life, ranging from archaea to mammals [1]. Based on structural characteristics and functional properties, peroxidases can be classified into haem peroxidase and non-haem peroxidase [2]. Haem peroxidases, which are characterized by the presence of a prosthetic heme group, are further subdivided into animal-type and non-animal-type subgroups. Notably, non-animal haem peroxidases are further classified into three distinct classes: Class I peroxidases (intracellular enzymes), Class II peroxidases (extracellular fungal enzymes), and Class III peroxidases (plant secretory peroxidases) [3]. Class III peroxidases (EC 1.11.1.7) are plant-specific oxidoreductases, also known by several abbreviations, including PER, POD, POX, Prx, and Px [4,5]. To maintain nomenclatural consistency, we refer to them exclusively as POD throughout this study. It is important to note that peroxiredoxins (Prx; EC 1.11.1.15), despite some overlapping abbreviations, represent an evolutionarily distinct protein family, and are not included in our analysis.

The functions of the *POD* genes have been extensively characterized in numerous studies, demonstrating their crucial roles in plant growth, development, and responses to various stresses. For instance, *PODs* have been documented to participate in a wide range of biological processes, including seed germination [6], root elongation [7], pollen development [8], fruit ripening [9], cell wall metabolism [10], lignification [11,12], reactive oxygen species (ROS) generation and scavenging [13,14], and phytohormone metabolism [15,16]. In addition, *PODs* play pivotal roles in plant defense against diverse abiotic stressors, such as drought [17], salinity [18], and low temperature [19], and biotic stressors, such as bacterial and fungal pathogens [20,21]. Notably, *PODs* have been implicated in the regulation of the anthocyanin metabolism, particularly in the degradation of anthocyanins [22]. In *Brunfelsia calycina*, *BcPOD1* was presented in the vacuoles of deeply purple pigmented petals, where its expression led to the degradation of anthocyanin [23]. In the strawberry, *FaPOD27* has been closely associated with anthocyanin degradation in the fruit [24]. High temperatures have been shown to induce the expression of *PODs* in the grapevine and in *Malus profusion*, leading to enhanced anthocyanin degradation [25,26].

The radish (*Raphanus sativus* L.) is an important root vegetable crop, extensively cultivated across Asia, Europe, and North America [27]. Through prolonged natural selection and artificial domestication processes, numerous radish cultivars with distinct taproot coloration have been systematically developed. Certain radish cultivars develop red or purple taproots owing to anthocyanin accumulation. Radish-derived anthocyanins exhibit remarkable thermal stability and potent anti-oxidant capacity, and have been extensively utilized as natural pigments in food processing, pharmaceutical manufacturing, and chemical industries. Therefore, research on radish anthocyanins has received growing attention [28,29]. The metabolic pathway of radish anthocyanins begins with the synthesis of the leucoanthocyanidin backbone using phenylalanine. Subsequently, this backbone undergoes glycosylation, and potentially methylation or acylation modifications, to form stable anthocyanins. The modified anthocyanins are subsequently transported into vacuoles for storage. In the acidic environment of the vacuole, anthocyanins exhibit different colors. Finally, polyphenol oxidase (PPO) or POD catalyze the oxidation and decomposition, completing the metabolic process. Currently, related genes for the synthesis and regulation of radish anthocyanins have been cloned [30,31], but research on the degradation of radish anthocyanins is still relatively scarce. The ‘Xinlimei’ radishes, a group of cultivars specific to China and distinguished by their red-to-purple root flesh and high anthocyanin content [32], serve as a valuable model for investigating the relationship between the anthocyanin metabolism and the POD activity. To date, the *POD* family members have been identified in numerous species, including 73 in Arabidopsis [33], 138 in rice [34], 91 in cassava [35], 102 in potato [36], 75 in carrot [37] and 109 in *Brassica napus* [38]. However, a genome-wide analysis of this gene family in the radish has not yet been reported.

This study presents the first comprehensive identification of all *POD* family members in the radish, accompanied by systematic analyses of their gene structure, evolutionary history, and functional diversification. A chimeric-colored radish mutant of ‘Xinlimei’ was employed to investigate the relationship between the anthocyanin metabolism and the POD activity in the taproot. Through a transcriptome analysis, a number of candidate *POD* genes potentially associated with anthocyanin degradation were identified. These findings establish a genome-wide framework of the *POD* members in the radish, and lay the foundation for subsequent functional studies on the specific *PODs* that may modulate the anthocyanin metabolism in this species.

## 2. Result

### 2.1. Identification and Characterization of the RsPODs

To comprehensively and accurately identify the *POD* genes in the radish, BLAST and hidden markov model (HMM)-based methods were employed using the previously reported Arabidopsis *POD* genes (*AtPODs*) as queries [33]. After filtering, 95 *POD* genes were identified from the radish genome and were designated as *RsPOD1* to *RsPOD95* based on their chromosomal positions (Figure S1). The *RsPODs* are unevenly distributed across nine chromosomes, with each chromosome containing 5 to 21 genes. Subsequently, the physicochemical properties of the RsPOD proteins were analyzed (Table S1). The RsPOD proteins range in length from 205 (RsPOD79) to 401 (RsPOD18) amino acids, with the molecular weight (MW) ranging from 22.35 to 44.56 kDa. The isoelectric point (pI) falls between 4.49 (RsPOD51) and 10.64 (RsPOD49), with 36 RsPODs classified as acidic proteins (pI < 7) and 59 as basic proteins (pI > 7). The grand average of hydropathicity (GRAVY) of most RsPODs was below 0, suggesting that the majority are hydrophilic proteins. Subcellular localization predictions revealed diverse cellular compartmentalization, with 41 RsPODs localized in the chloroplast, 25 in the extracellular space, 12 in the vascular membrane, 9 in the cytoplasm, 3 in the nucleus, 3 in the plasma membrane, and 2 in the endoplasmic reticulum. The structural diversity observed in the predicted three-dimensional models of the RsPOD proteinssuggests potential functional redundancy and sub/neo-functionalization among these genes in the radish (Figure S2).

### 2.2. Phylogenetic Relationship of the RsPODs

**Figure 1:**
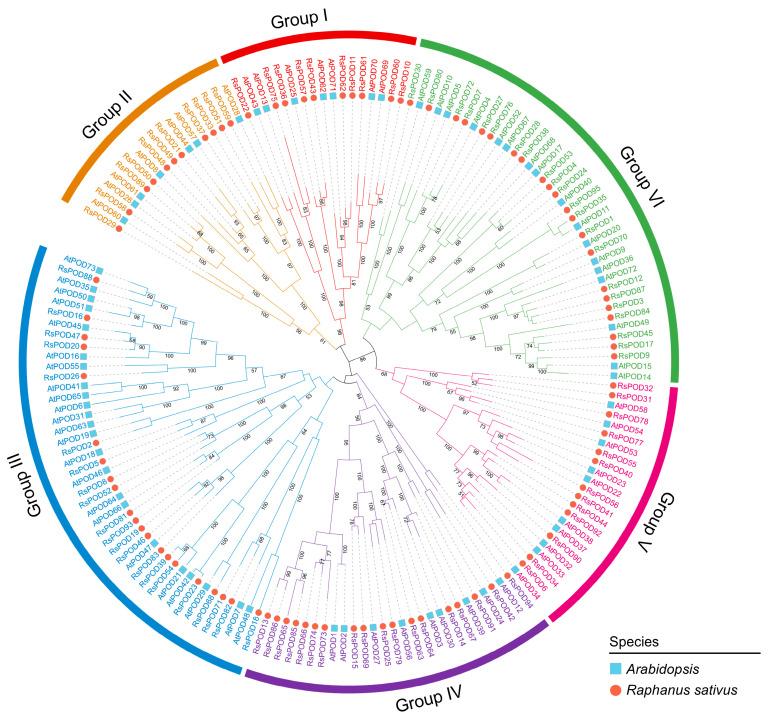
Phylogenetic analysis of POD proteins in *R. sativus* and *A. thaliana.* The PODs were divided into six subgroups (group I, II, III, IV, V, and VI). The blue squares and red circles represent the POD proteins in *A. thaliana* and *R. sativus*, respectively.

To further elucidate the evolutionary relationship of POD proteins, a maximum likelihood phylogenetic tree was constructed using MEGA11 software, incorporating 168 PODs from two species: 95 from *R. sativus* and 73 from *Arabidopsis thaliana*, based on their protein sequences. Based on the evolutionary divergence, the phylogenetic tree was divided into six subgroups (Figure 1). A phylogenetic analysis revealed that the POD proteins exhibited an uneven distribution across each subgroup. The largest subgroups, III and VI, consist of 21 and 22 RsPOD members, respectively, whereas the smallest subgroups, I and II, contain 10 and 11 RsPOD members, respectively. Subgroups IV and V are composed of 18 and 13 RsPOD members, respectively.

### 2.3. Motif and Structural Analysis of the RsPODs

**Figure 2:**
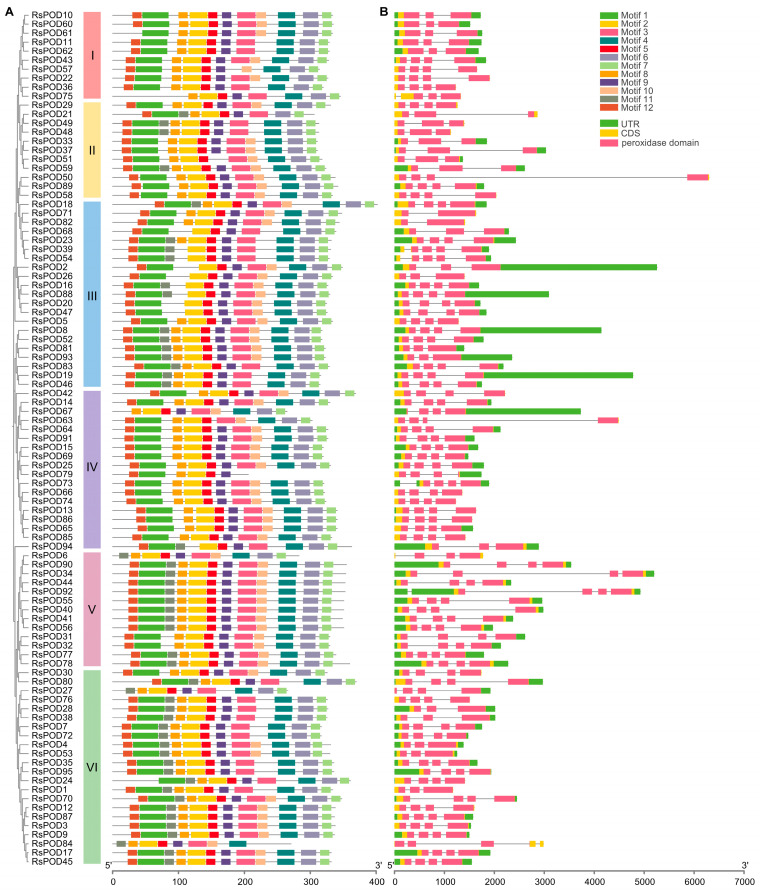
Conserved motifs and exon–intron structure analysis of the RsPODs. (**A**) The phylogenetic tree shows that the RsPODs are distributed in six groups (I–VI), which are indicated by red, yellow, blue, purple, pink, and green, respectively. Boxes with different colors represent different motifs. (**B**) The exon–intron structures of the *RsPODs*. The green, yellow, and pink boxes represent the UTR, CDS, and peroxidase domain, respectively. The size of the RsPOD members is indicated by the scales at the bottom.

Gene structure, coupled with their constituent motifs and domains, is often closely associated with gene function. To investigate the motif compositions among the RsPOD proteins, conserved motifs were analyzed using the Multiple EM for Motif Elicitation (MEME) online tool in accordance with the phylogenetic relationship, by which, a total of 12 conserved motifs were identified (Table S2). These conserved motifs were then annotated using the InterProScan 5 online platform. Four motifs (motifs 1–4) were identified as peroxidase domains, whereas the other motifs (motifs 5–12) had no known functional annotation. As shown in Figure 2A, most *RsPODs* within the same subgroup share similar motif composition and arrangement, with the exceptions including *RsPOD75* in subgroup I, *RsPOD67* and *RsPOD79* in subgroup IV, *RsPOD6* in subgroup V, and *RsPOD27* and *RsPOD84* in subgroup VI. To gain further insights into the evolutionary patterns of the *RsPOD* family, exon–intron structures in the *RsPOD* genes were analyzed. The gene structures and the location of the peroxidase domains are shown in Figure 2B. Most *RsPODs* contain three or four exons, while *RsPOD39*, *RsPOD54* and *RsPOD84* exhibit the most complex gene structures, each containing five exons. By contrast, *RsPOD71* and *RsPOD82* have the fewest exons, with only two. Additionally, *RsPOD73* and *RsPOD92* show intron insertion within the 5′ untranslated region (UTR) of the gene. In conclusion, the consistency of the phylogenetic relationships, the conserved motif patterns, and the gene structures supports the conserved gene organizations within the subgroups, and suggests that the *RsPOD* gene functions may have diversified and become more complex during evolution.

### 2.4. Collinearity Analysis of the RsPODs

**Figure 3:**
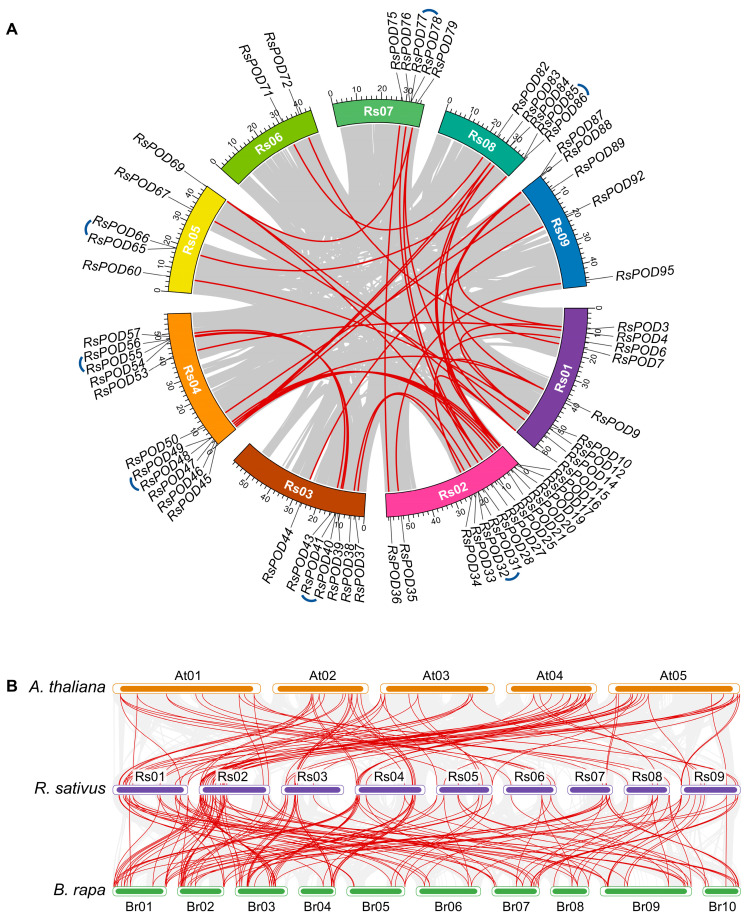
Collinearity analysis of the *RsPODs*. (**A**) Intraspecific collinearity analysis of the *RsPODs* in the radish. Different chromosomes are shown in different colors. Approximate positions of the *RsPODs* are marked with black lines on the chromosomes. Red and blue curves indicate segmental and tandem duplication events of the *RsPODs*, respectively. (**B**) Interspecific collinearity analysis of *PODs* in *A. thaliana*, *R. sativus*, and *B. rapa*. Red curves indicate the gene pairs of *PODs* in three species.

Plant genomes are frequently subject to gene family expansion events throughout evolutionary trajectories and domestication processes. To understand the expansion events of the *RsPODs* in the radish, an intraspecific collinearity analysis was conducted. Genome duplication events within *R. sativus* were investigated using TBtools-II software. As shown in Figure 3A, 47 collinear gene pairs were identified across 9 chromosomes with uneven distribution patterns, including 7 tandem and 40 segmental duplication events. For instance, *RsPOD31* and *RsPOD32* (*RsPOD40* and *RsPOD41*) likely originated from tandem duplication events, and *RsPOD15* and *RsPOD25* (*RsPOD33* and *RsPOD37*) likely originated from segmental duplication events. Chromosomes Rs01 and Rs02 contained the highest number of duplicated genes, suggesting that they play a major role in the expansion of the *RsPOD* family in the radish. Furthermore, the 47 *RsPOD* gene pairs were analyzed by calculating their non-synonymous (*Ka*) and synonymous (*Ks*). The substitution ratios (*Ka/Ks*) of all gene pairs were below 1 (Table S3), indicating these genes have undergone purifying selection during the evolution and domestication processes.

Syntenic relationships of the conserved chromosomal segments across divergent species provide critical evidence for elucidating the evolutionary origins of gene family members. To further comprehend the evolutionary dynamic of the *PODs* in the radish and other species, an interspecific collinearity analysis was conducted among three cruciferous plants (*A. thaliana*, *R. sativus*, and *Brassica rapa*). A total of 76 *RsPODs* were identified in 93 colinear blocks with 54 *A. thaliana* genes, and 79 *RsPODs* in 157 colinear blocks with 88 *B. rapa* genes (Figure 3B and Table S4). The greater number of collinear blocks between *B. rapa* and *R. sativus* compared to those between *A. thaliana* and *R. sativus* suggests a closer evolutionary relationship between *B. rapa* and *R. sativus*. Among these, 74 *RsPODs* were syntenic across all three species, suggesting these genes may play conserved and crucial roles throughout the evolution of cruciferous plants.

### 2.5. cis-Acting Elements in the RsPODs

**Figure 4:**
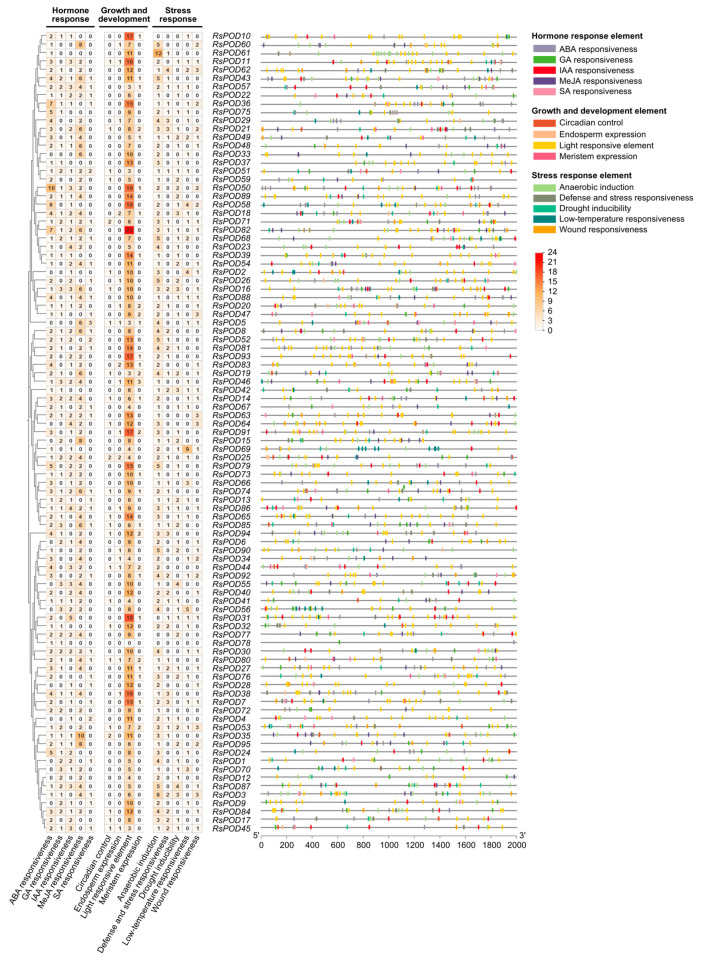
*cis*-element analysis in the promoter region of the *RsPODs*. The *cis*-elements were grouped according to their functional implications. The left heatmap shows the number of elements in each promoter. The distribution of these elements in the promoter region is shown on the right. Different elements are represented with different colored boxes.

*cis*-acting elements, located within the promoter region upstream of the gene transcription start site, are essential for gene expression. To explore the potential function of the *RsPODs*, a comprehensive analysis of *cis*-elements in the 2000 bp promoter region of the *RsPOD*s was conducted using the PlantCARE online platform. A total of 2172 *cis*-elements were predicted in the promoter region of the *RsPODs* (Table S5), and are visualized in Figure 4. Among them, *RsPOD82* contained the largest number of *cis*-elements (45), while *RsPOD78* had the least number of *cis*-elements (2). All *cis*-elements were classified into three major functional categories. The first category, the hormone response element (687), consisted of abscisic acid (ABA) responsiveness (192, 27.95%), gibberellin (GA) responsiveness (93, 13.54%), auxin (IAA) responsiveness (117, 17.03%), methyl jasmonate (MeJA) responsiveness (248, 36.10%), and salicylic acid (SA) responsiveness (37, 5.38%). Almost all *RsPOD* promoters contain multiple ABA responsiveness (ABRE) and MeJA-responsiveness (CGTCA-motif and TGACG-motif), suggesting that they may respond to specific hormones in a rapid and robust way. The second category, the growth and development element (976), consisted of circadian control (28, 2.87%), endosperm expression (21, 2.15%), light responsive element (887, 90.88%), and meristem expression (40, 4.10%). Light responsive elements (e.g., ACE, GT1-motif, Box 4) were highly abundant in nearly all *RsPOD* promoters, while circadian control (circadian), endosperm expression (GCN4_motif), and meristem expression (CAT-box) were relatively scarce. The third category, the stress response element (509), consisted of anaerobic induction (231, 45.38%), defense and stress responsiveness (69, 13.56%), drought inducibility (84, 16.50%), low-temperature responsiveness (63, 12.38%), and wound responsiveness (62, 12.18%). Anaerobic induction elements (ARE) were ubiquitously present in the *RsPOD* promoters, while defense and stress responsiveness (TC-rich repeats), drought inducibility (DRE core and MBS), low-temperature responsiveness (LTR), and wound responsiveness (WUN-motif) were present in only certain members. For instance, *RsPOD62* contained four drought inducibility elements and *RsPOD69* had nine low-temperature responsiveness elements, suggesting they play distinct roles in stress responses. These findings suggest that the *RsPODs* play critical roles in plant growth and development, hormone signaling, and stress tolerance.

### 2.6. Prediction Analysis of the RsPOD-Mediated Regulatory Network

**Figure 5:**
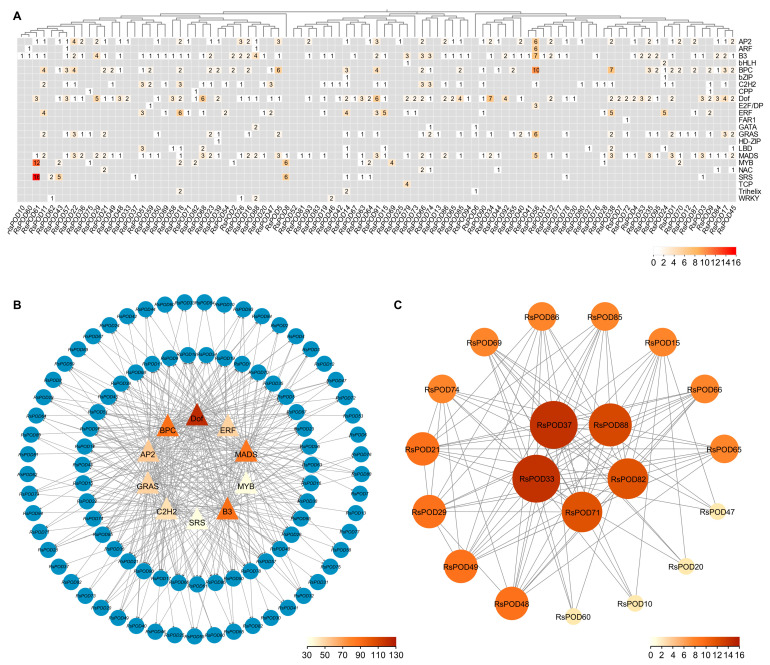
Regulatory network of the RsPODs. (**A**) Heatmap showing the number of TF binding sites in promoter regions of the *RsPODs*. (**B**) The top 10 highly enriched TFs and their targeted *RsPODs*. Triangle and circle nodes represent TFs and their targeted *RsPODs*, respectively. The node color represents the number of TFs. (**C**) Functional interaction networks of the RsPOD proteins. The node color represents the number of proteins.

PODs are typically regulated by transcription factors (TFs) and interact with other proteins to execute their physiological roles. To elucidate the transcriptional regulatory networks and underlying mechanisms governing the RsPODs in the radish, we systematically predicted TF binding sites within the promoter regions of the *RsPOD* genes using the PlantRegMap online tool. A subsequent analysis identified 748 binding sites corresponding to 23 distinct TF families, representing the putative regulatory elements of the *RsPODs* (Table S6). As shown in Figure 5A, *RsPOD56* contained the highest number of regulatory sites (44), followed by *RsPOD61* (34), *RsPOD38* (24), and *RsPOD91* (23). Furthermore, we prioritized the top 10 TF families based on interaction frequency, including the Dof (122), B3 (90), BPC (90), MADS (85), AP2 (75), GRAS (54), ERF (51), C2H2 (46), MYB (36), and SRS (35) families, and mapped their predicted regulatory interactions with specific *RsPODs* (Figure 5B). These TF families exhibited significant enrichment, suggesting their putative regulatory roles in modulating *RsPOD* transcription. In addition, a protein–protein interaction (PPI) network for the RsPOD proteins was constructed using the STRING database (Figure 5C). The RsPODs appeared to function by forming heterodimers with other members within their family, but no interactions were observed with the protein from other families. Notably, RsPOD33 and RsPOD37 exhibited the highest number of interactions with other RsPODs (16), whereas RsPOD10, RsPOD20, RsPOD47 and RsPOD60 were found to exclusively interact with only 4 distinct RsPODs.

### 2.7. Expression Profiles of the RsPODs in Different Tissues

**Figure 6:**
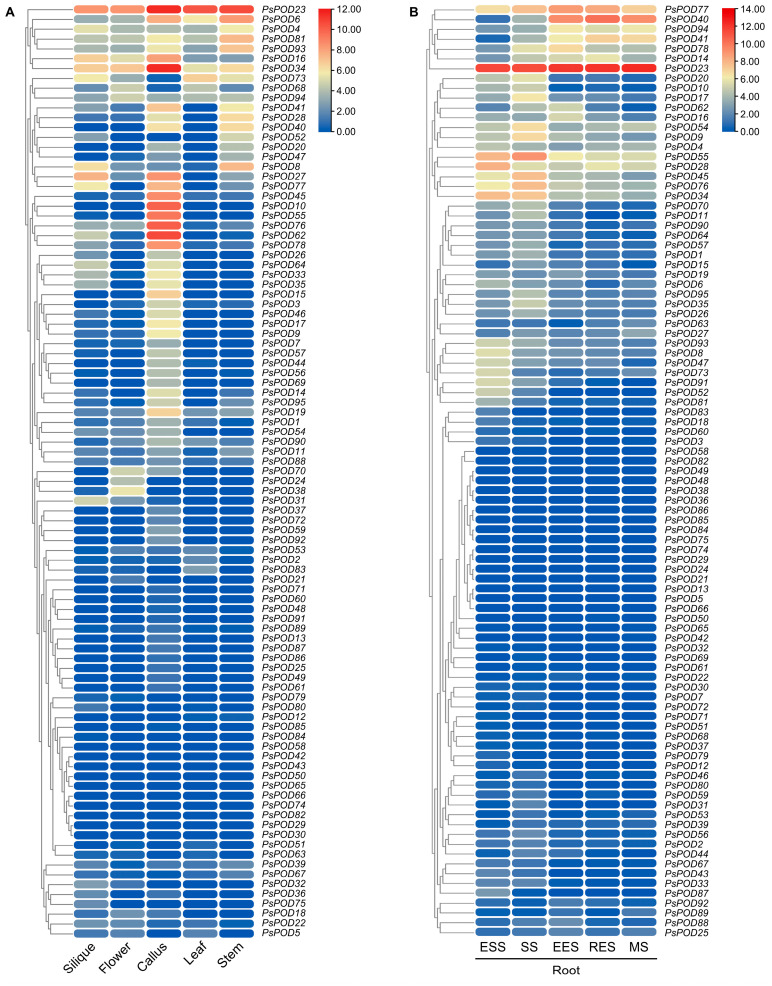
Expression analysis of the *RsPODs* in different tissues based on RNA-seq data. (**A**) Expression analysis of the *RsPODs* in five tissues, including the silique, flower, callus, leaf, and stem. (**B**) Expression analysis of the *RsPODs* in five root developmental stages. ESS, seedling stage; SS, splitting stage; EES, early expanding stage; RES, rapid expanding stage; MS, mature stage. The expression levels are calculated by log_2_ (FPKM + 1) values.

To explore the potential biological functions of the *RsPODs* in the radish, their expression patterns in five different tissues and five representative developmental stages of roots were analyzed using RNA-seq data from the previous studies [32,39]. A heatmap was constructed based on the fragments per kilobase of transcript per million fragments mapped reads (FPKM) values of the *RsPODs* (Figure 6). The *RsPODs* showed differential expression across various tissues and developmental stages. Among them, *RsPOD23* was highly expressed across all tissues and stages, while *RsPOD29* and *RsPOD66* were not expressed in any. In addition, several *RsPODs* exhibited tissue- or stage-specific expression patterns. For instance, *RsPOD24*, *RsPOD38*, and *RsPOD70* were highly expressed in the flower; *RsPOD10*, *RsPOD45*, *RsPOD55*, and *RsPOD76* were highly expressed in the callus; *RsPOD34*, *RsPOD45*, *RsPOD55*, and *RsPOD76* were highly expressed in the ESS and SS stages; *RsPOD40*, *RsPOD41*, and *RsPOD94* were highly expressed in the EES, RES and SS stages. These findings suggest that the *RsPODs* display diverse expression patterns, indicating their important roles in radish development.

### 2.8. RNA-Seq Analysis in the Radish

**Figure 7:**
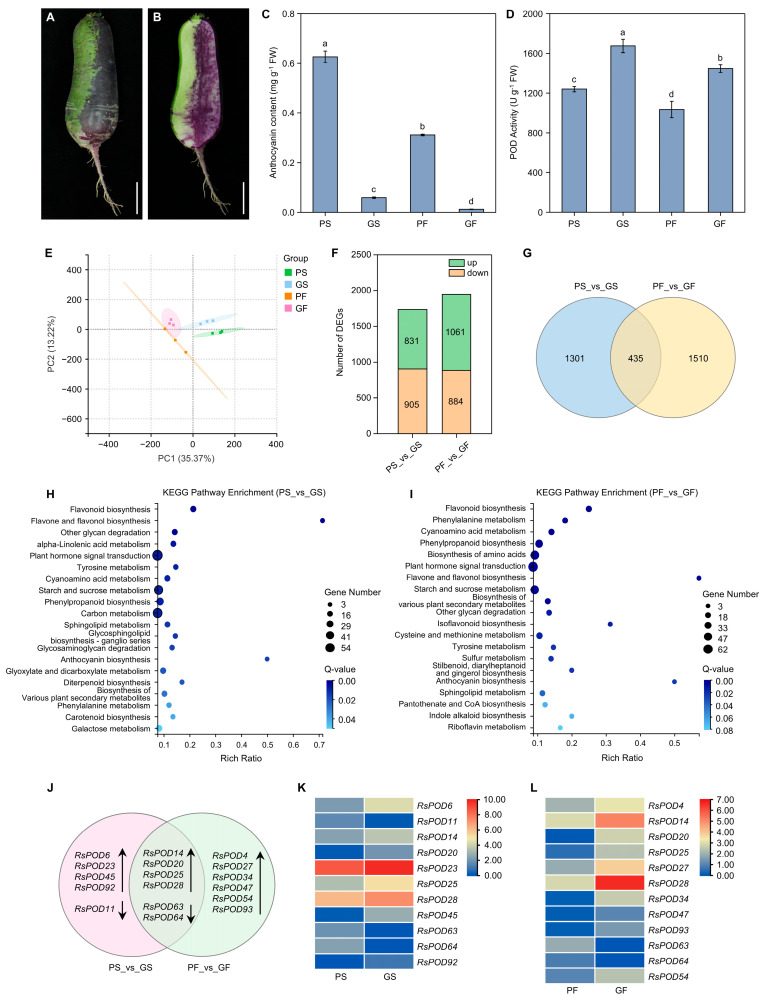
Transcriptome analysis of the chimeric-colored radish. (**A**,**B**) Phenotype of the chimeric-colored radish taproot. Scale bar = 5 cm. (**C**,**D**) The anthocyanin content and POD activity in four different tissues. Data are presented as a mean ± SD (n = 3). Different letters indicate statistically significant differences (one-way ANOVA followed by post hoc Tukey test; *p* < 0.05). PS, purple skin; GS, green skin; PF, purple flesh; GF, green flesh. (**E**) PCA of all genes identified from the PS, GS, PF, and GF. (**F**) The number of DEGs in the PS vs. GS and the PF vs. GF. (**G**) Venn diagrams of the DEGs of cross-comparisons between the PS vs. GS and the PF vs. GF. (**H**,**I**) KEGG enrichment analysis of the DEGs in the PS vs. GS and the PF vs. GF comparison, respectively. (**J**) Venn diagram of differentially expressed *RsPODs* found in the different tissues. (**K**,**L**) Heatmap shows the expression of differentially expressed *RsPODs* in skin and flesh, respectively. The expression levels are calculated by log2 (FPKM + 1) values.

To investigate the anthocyanin metabolism in the radish, a chimeric-colored radish mutant derived from a purple skin and purple flesh ‘Xinlimei’ cultivar was selected for further analysis. This mutant exhibited a dichotomous phenotype, with half of the root skin and flesh tissue displaying purple pigmentation and the other half showing green pigmentation (Figure 7A,B). Comparative analyses were conducted to measure the anthocyanin content and POD enzyme activity across distinct tissue sectors. The results demonstrate that the purple skin (PS) and purple flesh (PF) sectors contained significantly higher anthocyanin content compared to the green skin (GS) and green flesh (GF) (Figure 7C). Conversely, the POD activity levels in the PS and PF were markedly lower than those detected in the GS and GF tissues (Figure 7D).

Subsequently, an RNA-seq analysis was conducted. The root skin and root flesh with different colors were collected separately. Samples were obtained from three individual plants separately, and subjected to independent transcriptome sequencing. Each sample generated over 2.0 Gb of clean reads per sample group. More than 89% of the bases had Phred quality scores above 30 (Q30), and the alignment rate to the reference genome exceeded 94%, confirming high sequencing quality and reliability for downstream processes (Table S7). A principal component analysis (PCA) revealed distinct clustering of the PS, GS, PF, and GF, with PC1 and PC2 accounting for 35.37% and 13.22% of the variance, respectively (Figure 7E). A total of 1736 differentially expressed genes (DEGs) were identified between the PS and the GS, including 831 upregulated and 905 downregulated genes. The comparison between the PF and the GF revealed 1945 DEGs, comprising 1061 upregulated and 884 downregulated genes (Figure 7F). Notably, 435 common DEGs were shared between these two comparative pairs (Figure 7G and Table S8). Then, a KEGG pathway enrichment analysis was performed to identify the functional roles of the DEGs in both comparative pairs. As shown in Figure 7H,I, PS vs. GS and PF vs. GF exhibited similar metabolic patterns. In the PS vs. GS comparison, the most enriched pathways were the plant hormone signal transduction, the carbon metabolism, and the starch and sucrose metabolism, with flavone and flavonol biosynthesis, anthocyanin biosynthesis, and flavonoid biosynthesis displaying the highest rich ratios. Similarly, in the PF vs. GF comparison, the DEGs were most enriched in the plant hormone signal transduction, the biosynthesis of amino acids, and the starch and sucrose metabolism, with flavone and flavonol biosynthesis, anthocyanin biosynthesis, and isoflavonoid biosynthesis showing the highest ratios.

### 2.9. Potential Roles of the RsPODs in the Anthocyanin Metabolism

To explore the regulatory role of the *RsPOD* genes in the anthocyanin metabolism in the radish, we identified the *RsPODs* among the DEGs of two comparative pairs from the RNA-seq data. In the PS vs. GS comparison, eleven differentially expressed *RsPOD* genes were detected, with eight exhibiting upregulation in the GS (Figure 7J,K). In the PF vs. GF comparison, twelve *RsPOD* genes were identified as DEGs, with ten upregulated in the GF (Figure 7I,J). Six *RsPOD* genes were common DEGs in both comparisons; among these, four (*RsPOD14*, *RsPOD20*, *RsPOD25*, and *RsPOD28*) were upregulated in both the GS and the GF. These results indicate that most differentially expressed *RsPOD* genes exhibited higher expression levels in the green tissues than in the purple tissues. Notably, *RsPOD14*, *RsPOD20*, *RsPOD25*, and *RsPOD28* were potentially involved in anthocyanin degradation in both the skin and the flesh tissues.

### 2.10. Co-Expression Network of the RsPODs in the Anthocyanin Metabolism

**Figure 8:**
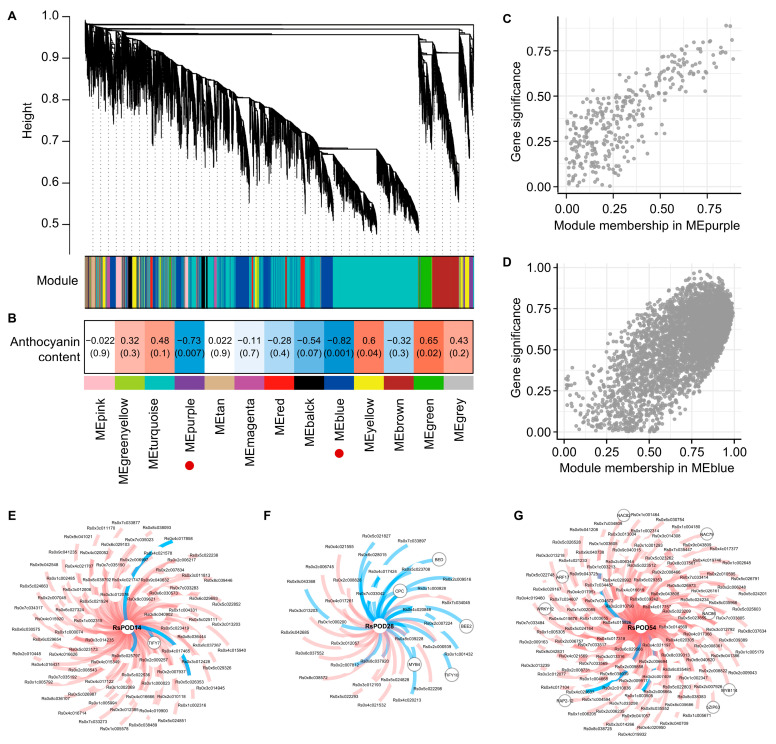
Co-expression network analysis of the *RsPODs* and the related genes based on the RNA-seq data. (**A**) Hierarchical cluster tree revealing gene co-expression modules identified by the WGCNA. The branches contain 13 modules labeled in different colors. (**B**) Module-trait associations. The correlation between the two is shown in the cell by the Spearman correlation coefficient, and the *p*-value is in parentheses. Cell color ranges from red (high positive correlation) to blue (high negative correlation). The red circles indicate that these module have a significant negative correlation with the anthocyanin content. (**C**,**D**) Correlation analysis between module membership (MM) and gene significance for genes in the MEpurple and MEblue, respectively. For each grey (unsorted) gene point, absolute values were computed for both MM and gene significance. (**E**–**G**) Co-expression network of *RsPOD14* and *RsPOD28* in MEpurple, as well as *RsPOD54* in MEblue, respectively. Transcription factors are encircled within gray circles, with red lines indicating positive correlations and blue lines indicating negative correlations.

To elucidate the co-expression network between the *RsPOD* genes and the related genes, a weighted gene co-expression network analysis (WGCNA) was performed using the RNA-seq data from the chimeric-colored radish mutants. After filtering, 20,877 genes were retained and grouped into 13 modules (MEs), comprising 12 distinct co-expression networks. The module sizes ranged from 244 (MEtan) to 9740 (MEturquoise), with outliers assigned to the unclustered MEgrey module (Figure 8A). To identify the modules significantly associated with anthocyanin content, the module-trait correlations were analyzed (Figure 8B). Anthocyanin content exhibited significant negative correlations with MEpurple (*R* = −0.73) and MEblue (*R* = −0.82). *RsPOD14* and *RsPOD28* were found to belong to MEpurple, while *RsPOD54* was assigned to MEblue (Figure 8C,D). Notably, *RsPOD14* and *RsPOD28* were common DEGs in both the PS vs. GS and the PF vs. GF comparisons, while *RsPOD54* was a DEG in the PF vs. GF comparison (Figure 7J). Genes co-expressed with these three *RsPODs* (Spearman correlation coefficient, |SCC| > 0.80) were screened and visualized for further analysis (Table S9). As shown in Figure 8E–G, one TF (*TIFY7*) is co-expressed with *RsPOD14,* five TFs (*BED*, *BEE2*, *CPC*, *MYB4*, and *TIFY10*) are co-expressed with *RsPOD28*, and eight TFs (*ARF17*, *bZIP63*, *MYB114*, *RAP2-12*, *NAC79*, *NAC82*, *NAC86*, and *WRKY12*) are co-expressed with *RsPOD54*. These results indicate that *RsPOD14*, *RsPOD28*, and *RsPOD54* are closely associated with anthocyanin degradation. The TFs co-expressed with these *RsPOD* genes may participate in the anthocyanin metabolism by regulating their expression.

### 2.11. RT-qPCR Analysis of the RsPODs

**Figure 9:**
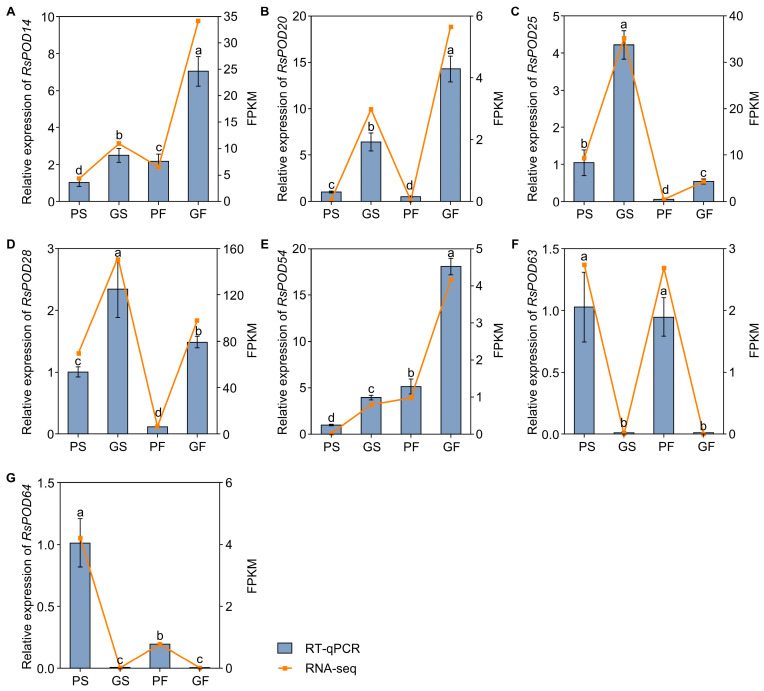
Validation of the selected *RsPODs* by RT-qPCR. (**A**–**G**) The expression levels of selected *RsPODs* in different tissues. The radish *RsACTIN* gene was used as an internal control. The yellow broken line represents the FPKM of RNA-seq; the gray histogram represents the relative mRNA expression determined by qRT-PCR. The data are presented as the mean ± SD (n = 3). The different lowercase letters indicate statistically significant differences as determined by one-way ANOVA followed by Tukey’s post hoc test; *p* < 0.05.

To verify the reliability of the transcriptome data, seven *RsPOD* genes were selected to assess their expression levels by RT-qPCR. These genes included *RsPOD14*, *RsPOD20*, *RsPOD25*, *RsPOD28*, *RsPOD63*, and *RsPOD64*, which were identified as common DEGs in both the PS vs. GS and the PF vs. GF comparisons, as well as *RsPOD14*, *RsPOD28*, and *RsPOD54*, which were selected based on WGCNA. The RT-qPCR results showed that the expression patterns of these selected *RsPODs* were highly consistent with the RNA-seq data, thereby confirming the accuracy and reproducibility of the transcriptomic data (Figure 9). Subsequently, a correlation analysis was performed to investigate the relationship between the expression levels of the selected *RsPOD* genes and the POD enzyme activity (Table S10). The results showed that the POD enzyme activity was significantly positively correlated with the expression of *RsPOD20*, *RsPOD25*, and *RsPOD28*, whereas it was significantly negatively correlated with the expression of *RsPOD63*.

## 3. Discussion

Class III peroxidases exhibit multifunctional roles in plant metabolic and physiological processes [1,2,3]. *PODs* have been extensively characterized in diverse plant species, such as Arabidopsis [33], rice [34], cassava [35], potato [36], carrot [37], and *B. napus* [38]. However, the biological functions of numerous *RsPODs* and their underlying regulatory mechanisms remain largely unexplored. Therefore, a comprehensive genome-wide identification and an analysis of the *RsPOD* genes is imperative for enhancing our understanding of their functional roles and the molecular mechanisms in the radish. In this study, utilizing the reference radish genome published by Zhang et al. [27], we identified 95 *RsPODs*. By contrast, only 73 *POD* homologs have been characterized in Arabidopsis (Figure S1). A phylogenetic analysis classified POD proteins from both the radish and Arabidopsis into six distinct subgroups (Figure 1). The similar distribution of PODs across the subgroups in both species supports the notion of evolutionary conservation within the plant POD family.

Genomic structure is typically conserved during plant evolution and domestication [40]. In our study, as shown in Figure 2, the *RsPODs* displayed conserved motif compositions and gene structures. The majority of the *RsPODs* contain three to four exons; however, there are a few that exhibit variations in the intron–exon structure. For instance, *RsPOD39*, *RsPOD54*, and *RsPOD84* contain five exons, whereas *RsPOD71* and *RsPOD82* contain only two exons. These alterations in the exon–intron structure may endow these genes with unique functional capabilities, enabling their participation in a range of biological processes. Gene duplication is a main driver of gene evolution and expansion [41,42]. In the radish, we observed 7 tandem duplications and 40 segmental duplications (Figure 3A), indicating that segmental duplication is the primary factor contributing to the expansion of the *RsPODs* in the radish. An analysis of the selection pressure on duplicated *RsPODs* suggests that they have undergone purifying selection during the evolution or domestication processes (Table S3). These findings are consistent with the results observed in maize [43], potato [36], Litchi [44], and *B. napus* [38]. Furthermore, 74 homologous *POD* genes were found to be conserved across *A. thaliana*, *R. sativus*, and *B. rapa* (Figure 3B and Table S4), suggesting that these genes possess similar functions and regulatory mechanisms, and play a pivotal role in the evolutionary trajectory of the *POD* family.

*cis*-acting elements located in the promoter region are closely linked to gene function [45,46]. In the promoter region of the *RsPODs*, we identified *cis*-elements involved in hormone responses, growth and development, as well as defense and stress responses (Figure 4), highlighting the functional complexity of the *RsPODs*. Although most *RsPODs* share conserved gene structures and motifs, their *cis*-elements exhibit distinct variations, possibly due to long-term adaptation to environmental changes [47]. TFs regulate the transcription of downstream genes by binding to specific sites within the promoter region [48]. In our study, as shown in Figure 5A, a substantial number of TF binding sites were identified in the promoter regions of the *RsPODs*. Among these, Dof, B3, BPC, MADS, AP2, GRAS, ERF, C2H2, MYB, and SRS exhibited the highest binding densities (Figure 5B), suggesting these TFs play critical regulatory roles in modulating *RsPOD*-mediated physiological processes. Certain genes, such as *RsPOD56*, *RsPOD61*, *RsPOD38*, and *RsPOD91*, possess a large number of binding sites in their promoters, indicating more complex transcriptional regulatory mechanisms [49]. The expression profiles of the *RsPODs* across different tissues and developmental stages of the radish revealed spatiotemporally specific expression patterns (Figure 6), indicating their sustained activity throughout the radish development.

Peroxidases are known to participate in the anthocyanin metabolism, particularly in degradation processes [22]. However, the role of the *RsPOD* genes in the degradation of anthocyanins in the radish remains poorly understood. In our study, we utilized a chimeric-colored radish mutant derived from a purple skin and purple flesh ‘Xinlimei’ cultivar (Figure 7A,B). Due to its distinct green and purple taproot sectors, this mutant serves as an ideal model for studying the relationship between the anthocyanin metabolism and the POD activity. Our results found that, within the same tissue, the POD enzyme activity in the green parts was higher than the activity in the purple parts (Figure 7D), suggesting that elevated POD activity contributes to reduced anthocyanin accumulation in the radish. This observation is consistent with previous findings on the litchi, in which the reduced anthocyanin levels were associated with the increased POD activity during storage [50]. Moreover, the POD activity is closely associated with lignin biosynthesis, as peroxidases catalyze the final step of lignin formation [51]. In Arabidopsis, the functional loss of *AtPOD2* or *AtPOD25* has been shown to lead to a significant reduction in lignin content [11]. In the carrot, xylem exhibiting lower anthocyanin content contains higher lignin levels than phloem with higher anthocyanin content [37]. In the strawberry, the metabolic interaction between anthocyanin synthesis and lignin formation is related to peroxidase, and the increased *FaPOD27* led to the transfer of anthocyanin to lignin in the strawberry fruits [24]. Consequently, the elevated POD enzyme activity observed in the green tissues of the radish (GS and GF) may potentially contribute to increased lignin accumulation, although further investigation is warranted to confirm this hypothesis.

A transcriptome analysis further revealed that four differentially expressed *RsPODs*, including *RsPOD14*, *RsPOD20*, *RsPOD25*, and *RsPOD28*, were upregulated in the green tissues compared to the purple tissues (Figure 7J). In addition, a WGCNA revealed that *RsPOD14*, *RsPOD28*, and *RsPOD54* were significantly negatively correlated with the anthocyanin content. Among them, *RsPOD14* and *RsPOD28* were present in both the differentially expressed *RsPODs* and the WGCNA result. Therefore, *RsPOD14* and *RsPOD28* may serve as key candidate genes involved in the anthocyanin metabolism. Future research will focus on elucidating the precise functions and regulatory mechanisms of these *RsPOD* genes, which will greatly enhance our understanding of the molecular basis of the anthocyanin metabolism in the radish.

## 4. Materials and Methods

### 4.1. Plant Materials

The chimeric-colored radish mutant of ‘Xinlimei’, exhibiting a taproot with green and purple sectors (both skin and flesh), was used in this study. The seeds were planted in late August in the experimental field of the Institute of Vegetables, Shandong Academy of Agricultural Sciences, and were harvested 70 days after sowing. Root skin and root flesh with different colors were collected separately. Samples were obtained from three individual plants separately, and subjected to independent downstream analyses. All materials were frozen in liquid nitrogen and stored at −80 °C for further study.

### 4.2. Identification of the RsPODs

The chromosome-level radish genome ‘Xinlimei’ (Rs00) were retrieved from the National Genomics Data Center (BioProject PRJCA003033; https://ngdc.cncb.ac.cn/gwh/Assembly/9797/show, accessed on 19 December 2024) [27]. Two complementary methods were used to identify the *POD* genes from the radish genome. First, a complete set of AtPOD protein sequences in Arabidopsis was obtained from the Tair database (https://www.arabidopsis.org/, accessed on 19 December 2024), and used as queries to retrieve the radish genome by the BlastP method with an *E*-value threshold of ≤1 × 10^−5^ in TBtools-II software v2.1.5 [52]. Second, HMM profiling was performed using the conserved peroxidase domain (PF00141) from the InterPro database (https://www.ebi.ac.uk/interpro/, accessed on 20 December 2024) [53]. Subsequently, all putative RsPOD members were validated using the online Conserved Domain Database (https://www.ncbi.nlm.nih.gov/cdd/, accessed on 21 December 2024) [54]. Finally, the identified *RsPOD* genes were renamed according to their physical positions in the radish genome.

### 4.3. Characterization of the RsPODs

The genetic information of the *RsPODs* was obtained from the radish genome database using TBtools-II software. The coding protein characteristics of the RsPODs, including protein length, MW, pI, instability index (II), aliphatic index (AI), and GRAVY, were calculated using the ProtParam tool on the Expasy server (https://web.expasy.org/protparam/, accessed on 5 January 2025) [55]. The subcellular localization of the RsPOD proteins were predicted using the WoLF PSORT website (https://wolfpsort.hgc.jp/, accessed on 5 January 2025) [56]. The three-dimensional structure of the RsPOD proteins was predicted and visualized using the AlphaFold 3 model (https://alphafoldserver.com/, accessed on 6 January 2025) and PyMOL software v2.6.2 [57].

### 4.4. Phylogenetic Analysis of the PODs

The full-length protein sequences from *R. sativus* and *A. thaliana* were employed to construct the phylogenetic tree using MEGA11 software v11.0.13 [58]. The alignment of the POD proteins was performed using the ClustalW method in MEGA11. A phylogenetic analysis of the aligned sequences was performed with the maximum likelihood method, WAG+G+I model, and 1000 bootstrap replicates. The generated phylogeny was optimized and visualized through the Interactive Tree of Life (iTOL) platform (https://itol.embl.de/index.shtml, accessed on 8 January 2025) [59].

### 4.5. Conserved Protein Motifs and Gene Structures Analysis of the RsPODs

The conserved motifs of the RsPOD proteins were analyzed using the MEME online tool (https://meme-suite.org/meme/tools/meme, accessed on 10 January 2025) [60], with the maximum number of motifs setting to 12. The other settings were kept at the default values. Then, these 12 motifs were annotated using the InterProScan 5 online platform (https://www.ebi.ac.uk/jdispatcher/pfa/iprscan5, accessed on 10 January 2025) [61]. The conserved domains of the RsPOD proteins were detected using the Batch CD-Search online tool (https://www.ncbi.nlm.nih.gov/Structure/bwrpsb/bwrpsb.cgi, accessed on 10 January 2025) [54]. The conserved protein motifs and gene structures combined with the phylogenetic tree were visualized using TBtools-II software.

### 4.6. Gene Duplication and Collinearity Analysis

The following analyses were conducted utilizing the TBtools-II software. The duplication events of the *RsPODs* were analyzed using the One Step MCScanX module. Gene location and gene linear relationship on the respective chromosomes were visualized using the Amazing Super Circos module. The evolutionary relationship between the *RsPODs* and tandem replication and segmental replication were calculated using the *Ka/Ks* Calculator module. A collinearity analysis among *R. sativus*, *A. thaliana*, and *B. rapa* were performed and visualized using the One Step MCScanX module and Multiple Synteny Plot module.

### 4.7. cis-Regulatory Element Analysis of the RsPODs

The 2000 bp sequence upstream of the translation start sites of the *RsPOD* genes was extracted from the radish genome using TBtools-II software, and regarded as the promoter region. The *cis*-elements in the promoter regions were analyzed and visualized using the PlantCARE website (https://bioinformatics.psb.ugent.be/webtools/plantcare/html/, accessed on 17 January 2025) and TBtools-II software [62].

### 4.8. The RsPOD-Mediated Regulatory Network

The TF binding sites in the promoter regions of the *RsPODs* were analyzed using the PlantRegMap online tool (https://plantregmap.gao-lab.org/binding_site_prediction.php, accessed on 20 January 2025) with a *p*-value ≤ 1 × 10^−5^ [63], and visualized using TBtools-II software. The PPI network of the RsPOD proteins was analyzed using the STRING website (https://cn.string-db.org/, accessed on 21 January 2025) [64]. Arabidopsis served as the selected plant species in the preceding analysis. The network map was constructed utilizing Cytoscape v3.10.0 software.

### 4.9. Determination of Total Anthocyanin Content and Peroxidase Activity

The total anthocyanin contents (ADS-W-KY016) and peroxidase activities (ADS-F-KY003) of the tissues with different colors were measured using assay kits (Jiangsu Aidisheng Biological Technology Co., Ltd., Yancheng, China). The analysis was performed with three independent biological replicates.

### 4.10. Transcriptome Sequencing and Data Analysis

The transcriptome data of different tissues and developmental stages (ESS: 11 days; SS: 21 days; EES: 44 days; RES: 56 days; MS: 73 days) were acquired from the NCBI Sequence Read Archive (SRA) repository (PRJNA353559 and PRJNA413464) [32,39]. Transcriptome sequencing of the tissues of the chimeric-colored radish mutant was completed by BGI (Beijing Genomics Institute, Shenzhen, China). Root skin and root flesh with different colors were collected separately. Samples were obtained from three individual plants separately and subjected to independent transcriptome sequencing. Briefly, the total RNA was extracted using the modified CTAB method and quantified using a fragment analyzer or an Agilent 2100 Bioanalyzer (Agilent, Santa Clara, CA, USA), or Qseq-400 (Bioptic, New Taipei, China). Library preparation was performed using an Optimal Dual-mode mRNA Library Prep Kit (BGI-Shenzhen, China). Paired-end 100/150 bases reads were generated on the G400 platform (BGI, Shenzhen, China).

After removing the adapter sequences and low-quality reads, the high-quality clean reads were mapped to the reference genome of the radish using the HISAT v2.1.0 [65]. The transcriptome was assembled and quantified using the StringTie v2.1.7 [66]. The values of FPKM were calculated based on gene lengths. Differential gene expression between the samples was calculated using DEseq2 v1.22.1 [67], based on the original count data. Genes with |log1.5 fold change| ≥ 1 and *Q*-value ≤ 0.05 were regarded as DEGs.

### 4.11. Expression Analyses

Total RNA was extracted from the tissues with different colors using the TransZol Up Kit (TransGen Biotech Co., Ltd., Beijing, China). An All-in-one First Strand cDNA Synthesis Kit Ⅱ (Sevenbio, Beijing, China) was used to synthesize cDNA from the total RNA. Reverse transcription quantitative PCR (RT-qPCR) was performed using a CFX96 Touch^TM^ real-time PCR system (Bio-Rad Laboratories, Hercules, CA, USA) and TB Green^®^ Premix Ex Taq™ II (RR82WR; Takara Biotechnology (Dalian) Co., Ltd., Dalian, China), according to the manufacturer’s protocol. The radish *RsACTIN* gene was used as the internal control. The expression levels were calculated using the comparative *C*_T_ method [68]. The analysis was performed with three independent biological replicates. The primers used for the expression analysis are listed in Table S11.

### 4.12. WGCNA Analysis

Transcriptome data from the chimeric-colored radish mutant and the WGCNA package were employed to construct a weighted gene co-expression network [69]. After filtering out genes with total FPKM values below 5 across all samples, normalizing the data via variance-stabilizing transformation (VST), and removing genes exhibiting zero median absolute deviation (MAD), the remaining genes were employed in subsequent analyses. The pickSoftThreshold function determined the soft power (β = 6) based on the scale-free topology criterion. An adjacency matrix was then generated using this soft threshold. Weighted co-expression modules were identified via the blockwiseModules function with the parameters power = 6, TOMType = unsigned, minModuleSize = 50, mergeCutHeight = 0.3, and the default settings for other parameters. Module-trait associations were assessed by calculating the Spearman correlation coefficients and *p*-values between module eigengenes and target traits. Within selected modules, pairwise correlations between the *RsPODs* and candidate genes were computed (Spearman correlation coefficient, |SCC| > 0.80), and the resulting co-expression network was visualized using Cytoscape software.

### 4.13. Statistical Analysis

All data were analyzed using one-way ANOVA with SPSS Statistics v30.0.0 (IBM, New York, NY, USA). Data are shown as a mean ± standard deviation.

## 5. Conclusions

In this study, 95 *RsPODs* were identified from the radish and their basic characteristics were systematically analyzed. Based on their domain architectures and phylogenetic relationships, these genes were classified into six distinct subgroups. A genomic collinearity analysis revealed that segmental and tandem duplication events contributed to the expansion of the *RsPOD* gene family, and a Ka/Ks analysis indicated that these genes have been subject to purifying selection during evolution and/or domestication. Further analyses of *cis*-elements, TF binding sites, and expression profiles demonstrated that the *RsPODs* likely participate in radish development and a range of physiological processes. Notably, a transcriptome analysis of the chimeric-colored radish mutant identified four *RsPODs* (*RsPOD14*, *RsPOD20*, *RsPOD25*, and *RsPOD28*) associated with the anthocyanin metabolism. In summary, our work establishes a theoretical foundation for functional investigations of the *RsPOD*s and provides candidate genes for elucidating the molecular mechanisms underlying the anthocyanin metabolism in the radish.

## Data Availability

All sequence data were deposited in the NCBI SRA under accession number PRJNA1255629.

## Supplementary Materials

The following supporting information can be downloaded at: https://www.mdpi.com/article/10.3390/ijms26135917/s1.
