# Supplementary material for: Genome-Wide Identification and Characterization of the Class III Peroxidase Gene Family in Radish (Raphanus sativus) with Insights into Their Roles in Anthocyanin Metabolism
Source: Int J Mol Sci. 2025 Jun 20;26(13):5917. doi: 10.3390/ijms26135917 (PMC12249633; doi:10.3390/ijms26135917)
Supplement: Supplementary file 1 [file ijms-26-05917-s001.zip › Supporting Figures.pdf]

# Supplementary Material

## Supplemental Figures S1-S2

**Article title:** Genome-Wide Identification and Characterization of the Class III Peroxidase Gene Family in Radish (Raphanus sativus) with Insights into Their Roles in Anthocyanin Metabolism

**Authors:** Zihao Wei†, Weimin Fu†, Xianxian Liu, Wenling Xu, Lichun Chang, Chen Liu*, Shufen Wang*

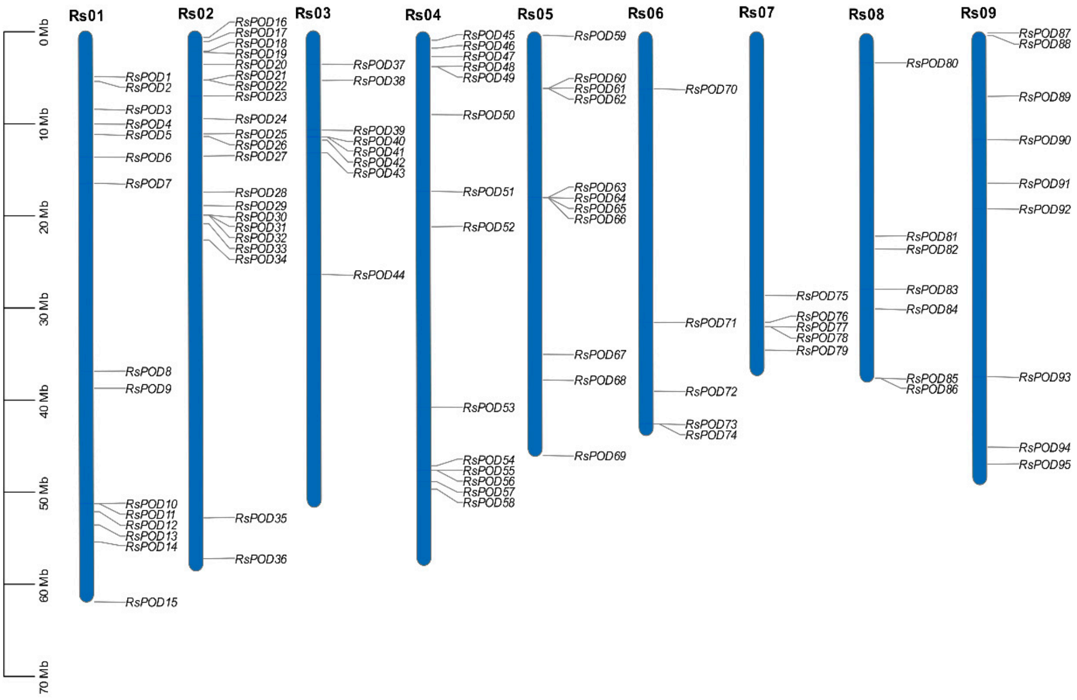


**Figure S1 Chromosomal distribution of the *RsPOD* genes.**

The distribution of the 95 *RsPODs* is based on relative physical positions.

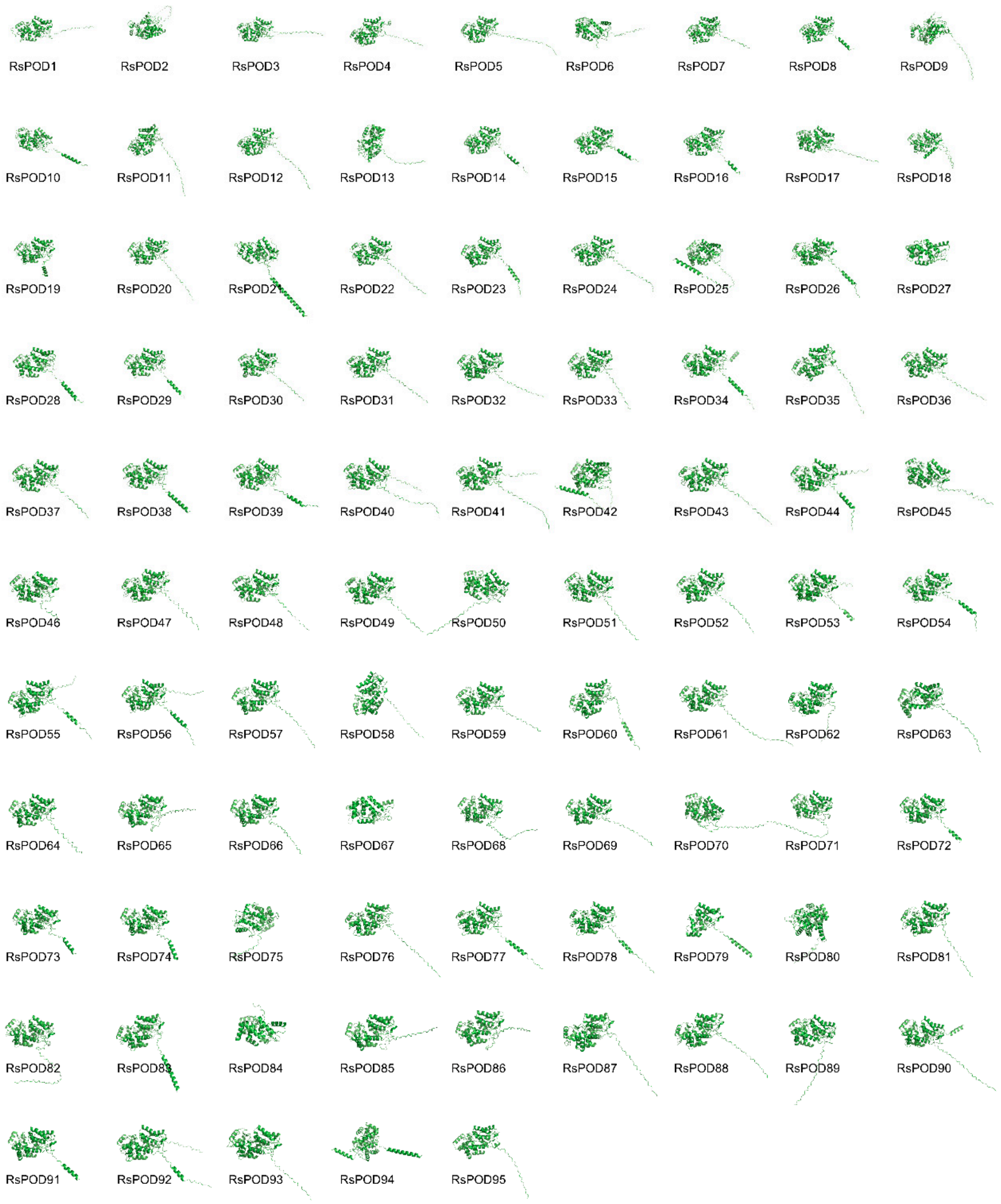


**Figure S2 Three-dimensional structure of the *RsPODs*.**
